# Survival outcomes with Lutetium-177-PSMA-617 in metastatic castration-resistant prostate cancer patient according to baseline tumor characteristics

**DOI:** 10.1007/s00345-026-06849-5

**Published:** 2026-10-20

**Authors:** Mike Wenzel, Quynh Chi Le, Carolin Siech, Benedikt Hoeh, Konrad Klimek, Christian Happel, Fred Saad, Felix Preisser, Derya Tilki, Markus Graefen, Thomas Steuber, Tobias Maurer, Maximilian C. Kriegmair, Pierre I. Karakiewicz, Felix K. H. Chun, Daniel Groener, Philipp Mandel

**Affiliations:** 1 Department of Urology, Goethe University Frankfurt, University Hospital, Frankfurt am Main, Germany; 2 Division of Urology, Cancer Prognostics and Health Outcomes Unit, University of Montréal Health Center, Montréal, Québec Canada; 3 Department of Urology, Carl Gustav Carus University Dresden, University Hospital, Dresden, Germany; 4 Martini-Klinik Prostate Cancer Center, University Hospital Hamburg- Eppendorf, Hamburg, Germany; 5 Department of Urology & Urosurgery, University Medical Centre Mannheim, Faculty of Medicine, University of Heidelberg, Mannheim, Germany; 6 Urological Hospital Munich-Planegg, Munich, Germany; 7 Department of Urology, University Hospital Hamburg-Eppendorf, Hamburg, Germany; 8 Department of Nuclear Medicine, Goethe University Frankfurt, University Hospital, Frankfurt am Main, Germany; 9 Department of Urology, Koc University Hospital, Istanbul, Turkey

**Keywords:** Lutetium-prostate-specific membrane antigen radioligand therapy, Survival outcomes, Baseline characteristics

## Abstract

**Purpose:**

To investigate cancer-control outcomes in metastatic castration-resistant prostate cancer (mCRPC) patients receiving Lutetium-177 Prostate-Specific Membrane Antigen-617 (Lu-177-PSMA) radioligand therapy according to initial baseline tumor characteristics.

**Methods:**

We relied on the FRAMCAP (FRAnkfurt Metastatic Cancer Database of the Prostate) database to assess progression-free survival (PFS) and overall survival (OS) in patients receiving Lu-177-PSMA as first to seventh mCRPC therapy line, according to initial time of metastasis (synchronous vs. metachronous), tumor grading (Gleason score [GS] 6–7 vs. 8–10) and M-stage (M1a vs. M1b vs. M1c) at initial metastatic hormone-sensitive prostate cancer (mHSPC). Kaplan-Meier curve analyses and multivariable Cox regression models were applied; multivariable models are additionally adjusted for ECOG status and line of Lu-177-PSMA therapy (1–2 L vs. 3 L vs. 4–7 L).

**Results:**

Of 344 Lu-177-PSMA mCRPC patients, 198 (58%) had synchronous vs. 146 (42%) metachronous mHSPC, 87 (29%) vs. 107 (62%) GS 6–7 vs. 8–10 and 15 (10%) vs. 121 (81%) vs. 13 (9%) M1a vs. M1b vs. M1c mHSPC disease. No significant differences were observed in PFS (12.1 vs. 13.0 months) or OS (14.9 vs. 18.3 months) between synchronous and metachronous patients (both p > = 0.09), or in PFS (12.9 vs. 11.8 months) and OS (21.4 vs. 15.1 months) between GS 6–7 and GS 8–10 (both p > = 0.3). M-stage-stratified analyses showed an OS difference (17.9 vs. 17.7 vs. 9.0 months for M1a vs. M1b vs. M1c, *p* = 0.01), but not for PFS (11.9 vs. 9.5 vs. 8.2 months, *p* = 0.08). After multivariable adjustment including ECOG status and treatment line, initial timing of metastasis, GS, and M-stage were not independently associated with PFS or OS. However, later treatment line was independently associated with worse OS (3 L vs. 1–2 L: HR 2.40, 95% CI 1.14–5.04, *p* = 0.021; 4–7 L vs. 1–2 L: HR 4.01, 95% CI 1.83–8.80, *p* < 0.001) as well as worse ECOG status (1–2 vs. 0: HR 2,60, 95% CI 1.38-4,91, *p* = 0.003).

**Conclusion:**

In this real-world cohort of mCRPC patients treated with Lu-177-PSMA, initial timing of metastatic disease, Gleason score, and M-stage were not independently associated with PFS or OS after multivariable adjustment including ECOG status and treatment line. These observational findings should not be interpreted as evidence of equivalent treatment efficacy across subgroups.

## Introduction

Metastatic castration-resistant prostate cancer (mCRPC) presents an advanced and often fatal stage of prostate cancer disease. However, over the past few decades, the therapeutic landscape has significantly evolved, allowing clinicians to achieve improved cancer control outcomes such as progression-free survival (PFS) and overall survival (OS), as demonstrated in recently published trials [1–10]. The approval of Lutetium-vipivotid-tetraxetan prostate-specific membrane antigen-617 radioligand therapy (Lu-177-PSMA) by the European Medical Agency in 2022, marks a notable advancement in the treatment sequence of mCRPC, particularly in patients with disease progression following standard mCRPC therapies such as taxan-based chemotherapy or androgen-receptor pathway inhibitors (ARPI) [8, 11].

Previous studies have highlighted the influence of certain baseline characteristics on survival outcomes in mCRPC, especially the prognostic significance of serum or molecular markers on Lu-177-PSMA outcomes [12–14]. Despite their established influence on survival outcomes in mCRPC patients, baseline factors such as Gleason Score (GS) [15, 16], tumor burden and location (M1a vs. M1b vs. M1c stage), as well as the initial timing of metastasis in metastatic hormone-sensitive prostate cancer (mHSPC) with either synchronous or metachronous metastasis have not been fully explored in the setting of Lu-177-PSMA radioligand therapy yet [17–20].

We addressed this void and relied on the FRAMCAP (Frankfurt Metastatic Cancer Database of the Prostate) database to investigate cancer-control outcomes such as PFS and OS in patients with mCRPC who received Lu-177-PSMA as first to seventh line mCRPC treatment with focus on various baseline characteristics. We hypothesized that important survival differences may exist according to baseline characteristics such as initial timing of metastasis in mHSPC, GS and M-stage in patients who underwent Lu-177-PSMA for mCRPC.

## Methods

### Study population

With approval from the local ethics committee (reference number: SUG-8–2024), we conducted a retrospective review of all mCRPC patients within the prospectively collected FRAMCAP database, adhering to the principles of the Declaration of Helsinki. The FRAMCAP database comprises of > 1200 patients with metastatic prostate cancer treated at the University Hospital Frankfurt, Germany, since 2014. All patients were assessed and discussed in a multimodal tumor board prior to and following treatment. For the analyses of the current study, we included patients with either synchronous or metachronous mHSPC prior to development of mCRPC and who were receiving Lu-177-PSMA between first and seventh lines of mCRPC treatment. These inclusion criteria yielded 344 patients, of which 198 had synchronous and 146 had metachronous mHSPC. Highest GS was assessed by samples from either prostate biopsy or radical prostatectomy. Staging and M-stage grouping at mHSPC was primarily performed using conventional imaging (CT-scan and bone scan) or PSMA-PET/CT. Patients with lymph nodes and bone metastases were grouped as M1b (bone metastases +/- lymph node metastases), patients with visceral metastases +/- bone +/- lymph nodes were grouped as M1c.

### Administration of Lu-177-PSMA radioligand therapy

Treatment of Lu-177-PSMA was administered at the department for nuclear medicine of the University Hospital Frankfurt, Germany, every 4–6 weeks, as previously described [14, 21–24] and patients were given either PSMA-617 or PSMA-I&T. Patients receiving Lu-177-PSMA were required to first, undergo PSMA-PET/CT scan and second, harbor PSMA-positive lesions. Lu-177-PSMA could be administered for patients as an individual compassionate use after previous case discussion in a multimodal tumor board or after its EMA approval post-ARPI and post-taxan-based chemotherapy.

### Statistical analysis

Descriptive characteristics included frequencies and proportions for categorical variables and median and interquartile ranges (IQR) for continuous variables. These were reported and tabulated in Table 1. The Chi-square test was used to determine statistical significance of differences in proportions, while t-test and Kruskal-Wallis-test were used to analyze differences in distributions. Kaplan-Meier curves depicted differences in PFS and OS according to synchronous vs. metachronous disease, GS (6–7 vs. 8–10), and M-stage (M1a vs. M1b vs. M1c). PFS was defined from initiation of Lu-177-PSMA treatment until initiation of a new systemic treatment or death; OS was defined from initiation of Lu-177-PSMA treatment until death from any cause. Univariable and multivariable Cox regression models were used to quantify associations with PFS and OS. Multivariable models included ECOG status, synchronous vs. metachronous mHSPC, GS, M-stage, and line of Lu-177-PSMA therapy, categorized as 1–2 L (reference), 3 L, and 4–7 L. All tests were two-sided with a significance level of *p* < 0.05. R software environment for statistical computing and graphics (version 4.4.3) was used for all analyses.

**Table 1.**
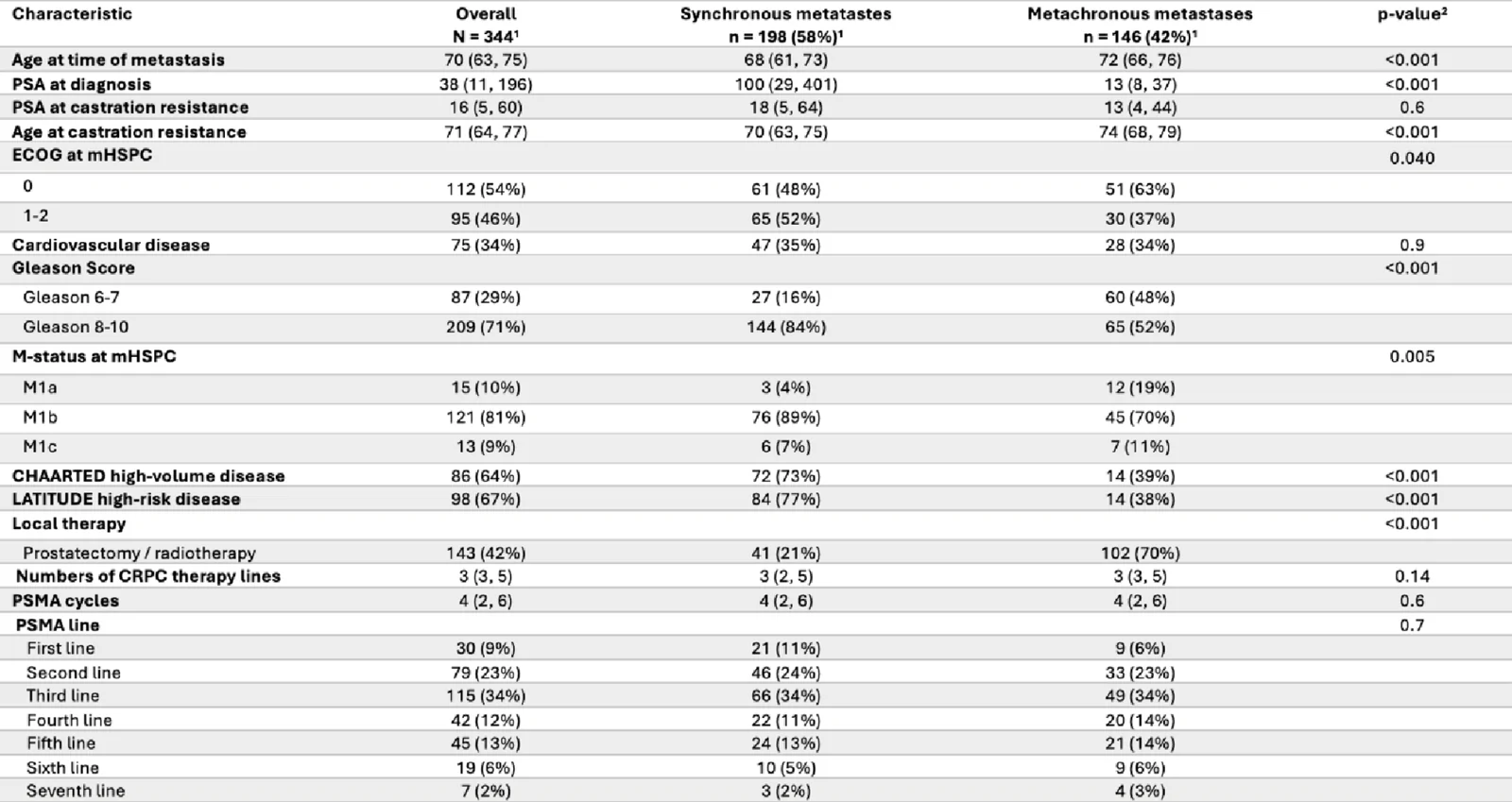
Baseline characteristics of 344 patients with either synchronous or metachronous metastatic hormone-sensitive prostate cancer patients undergoing 177-Lu-PSMA as first to seventh line after development of castration resistance

^1^Median (Q1, Q3); n (%), ^2^Kruskal-Wallis-rank sum test; Fisher’s exact test; Pearson’s Chi-square test

**Table 2.**
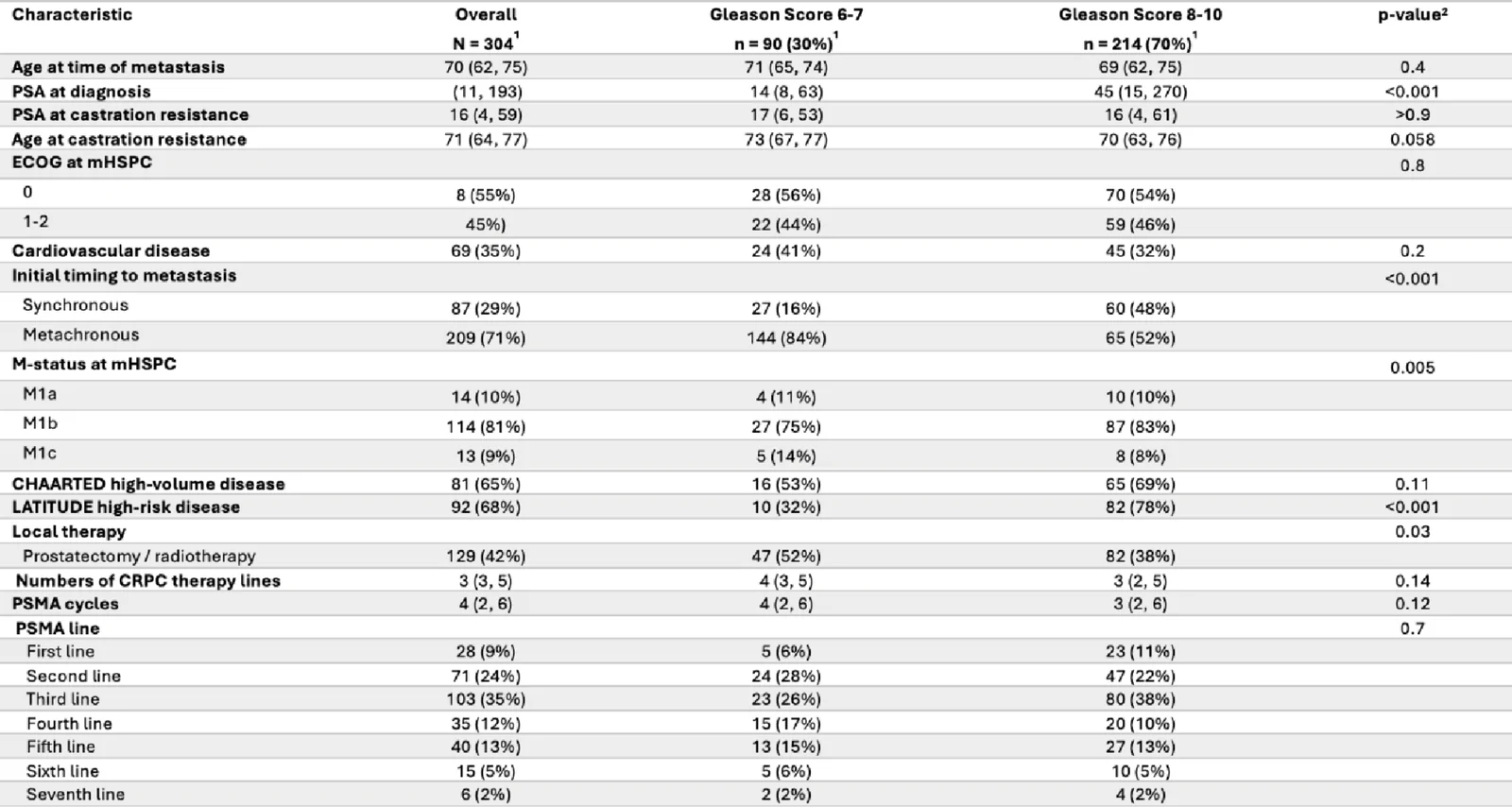
Baseline characteristics of 304 patients with metastatic castration-resistant prostate cancer patients undergoing 177-Lu-PSMA as first to seventh line according to Gleason Score

^1^Median (Q1, Q3); n (%), ^2^Kruskal-Wallis-rank sum test; Fisher’s exact test; Pearson’s Chi-square test

## Results

### Descriptive characteristics

Within 344 patients, median age at time of metastasis was 70 years (IQR 63, 75) and age at time of mCRPC was 71 years (IQR 64, 77). Median PSA at diagnosis was 38 ng/ml (IQR 11, 196), while PSA at mCRPC was 16 ng/ml (IQR 5, 60). Of 344 patients, 198 (58%) had synchronous and 146 (42%) metachronous metastatic prostate cancer (Table 1). Moreover, 87 (29%) vs. 209 (71%) harbored GS 6–7 vs. 8–10, while 15 (10%) vs. 121 (81%) vs. 13 (9%) had M1a vs. M1b vs. M1c stage at mHSPC (Table 1). According to stratification of synchronous vs. metachronous metastases, 86 (64%) patients harbored CHAARTED-high-volume [25] disease and 98 (67%) LATITUDE-high-risk disease [26]. Additionally, 112 (54%) harbored ECOG 0 status and 75 (34%) had cardiovascular disease (CVD). According to systemic therapy lines, median number of received therapy lines for mCRPC was 3 (IQR 3,5). Median number of PSMA cycles were 4 (IQR 2, 6) and the majority of patients received Lu-177-PSMA within the first five lines of therapy, with more than half who received it as second line (23%) or third line therapy (34%), while only 8% of the patients were administered to Lu-177-PSMA beyond the fifth line of mCRPC. Patients and tumor characteristics stratified according to synchronous vs. metachronous mHSPC, GS and M-stage are depicted in Tables 1, 2 and 3.

**Table 3.**
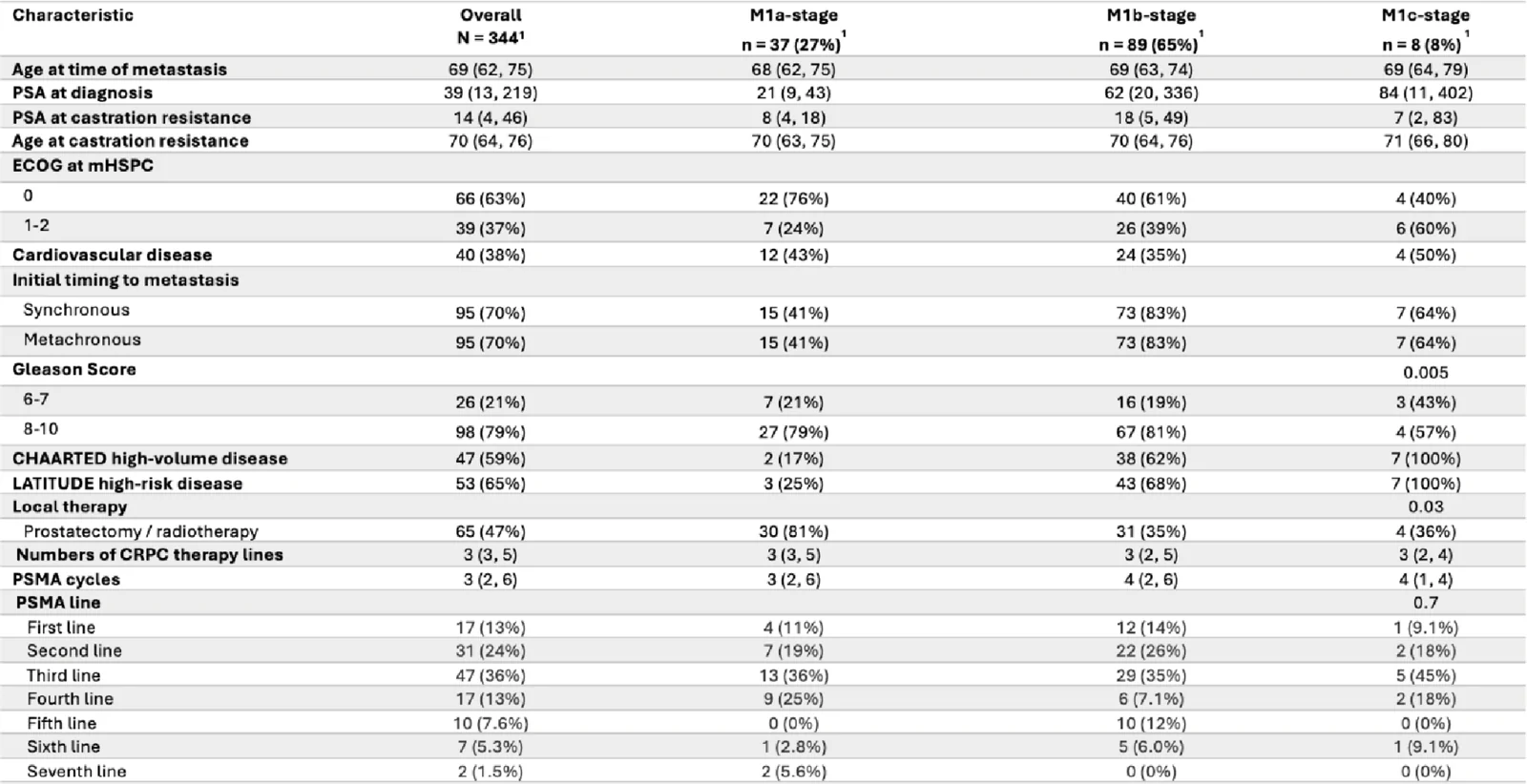
Baseline characteristics of 344 patients with metastatic castration-resistant prostate cancer patients undergoing 177-Lu-PSMA as first to seventh line according to M-stage

^1^Median (Q1, Q3); n (%), ^2^Kruskal-Wallis-rank sum test, Fisher’s exact test, Pearson’s Chi-square test

### PFS according to baseline characteristics

**Fig. 1:**
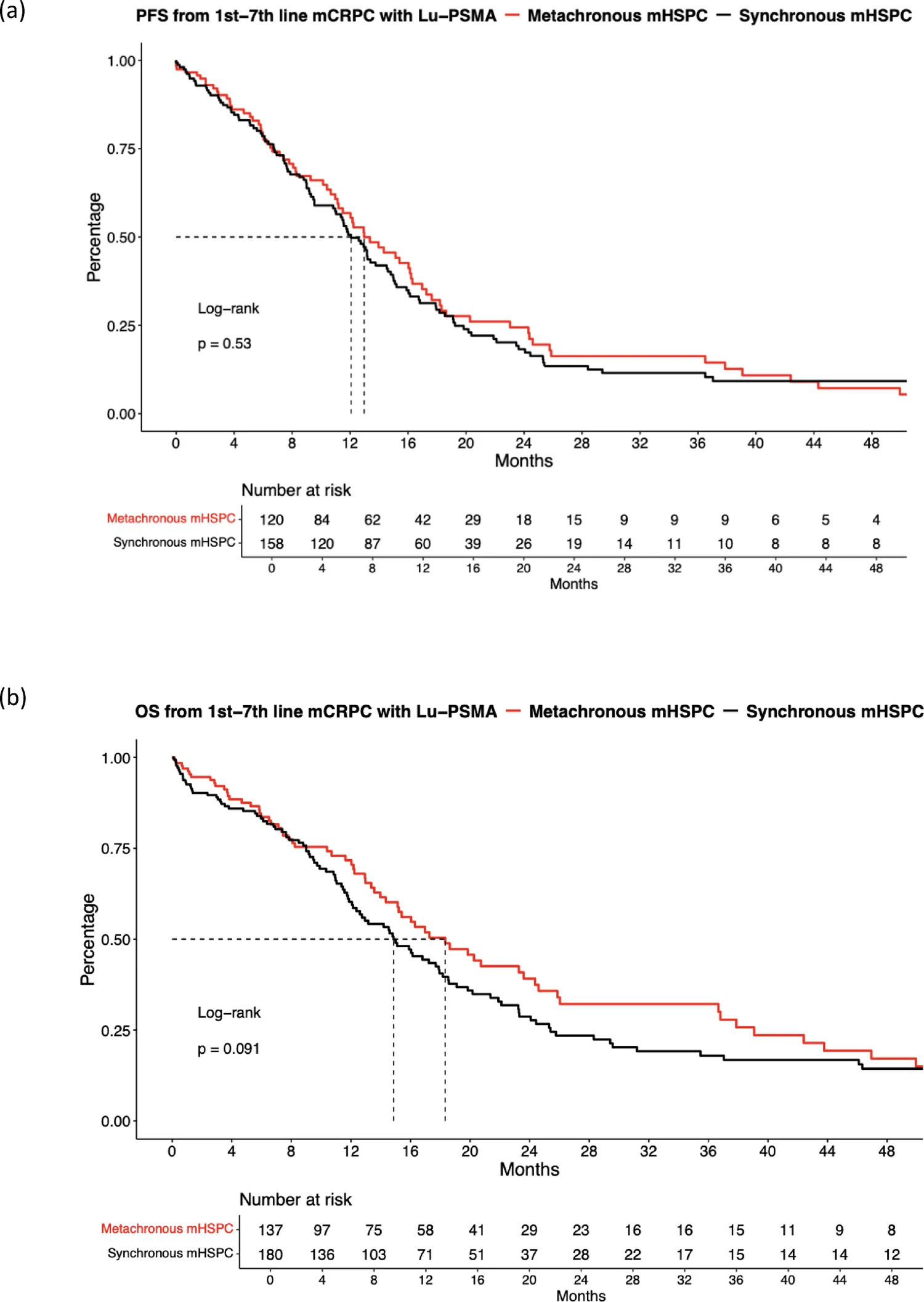
Kaplan Meier curves depicting **a** progression-free survival (PFS) and **b** overall survival (OS) with 177-Lu-PSMA therapy in metachronous vs. synchronous metastatic hormone-sensitive prostate cancer after castration resistance

**Fig. 2:**
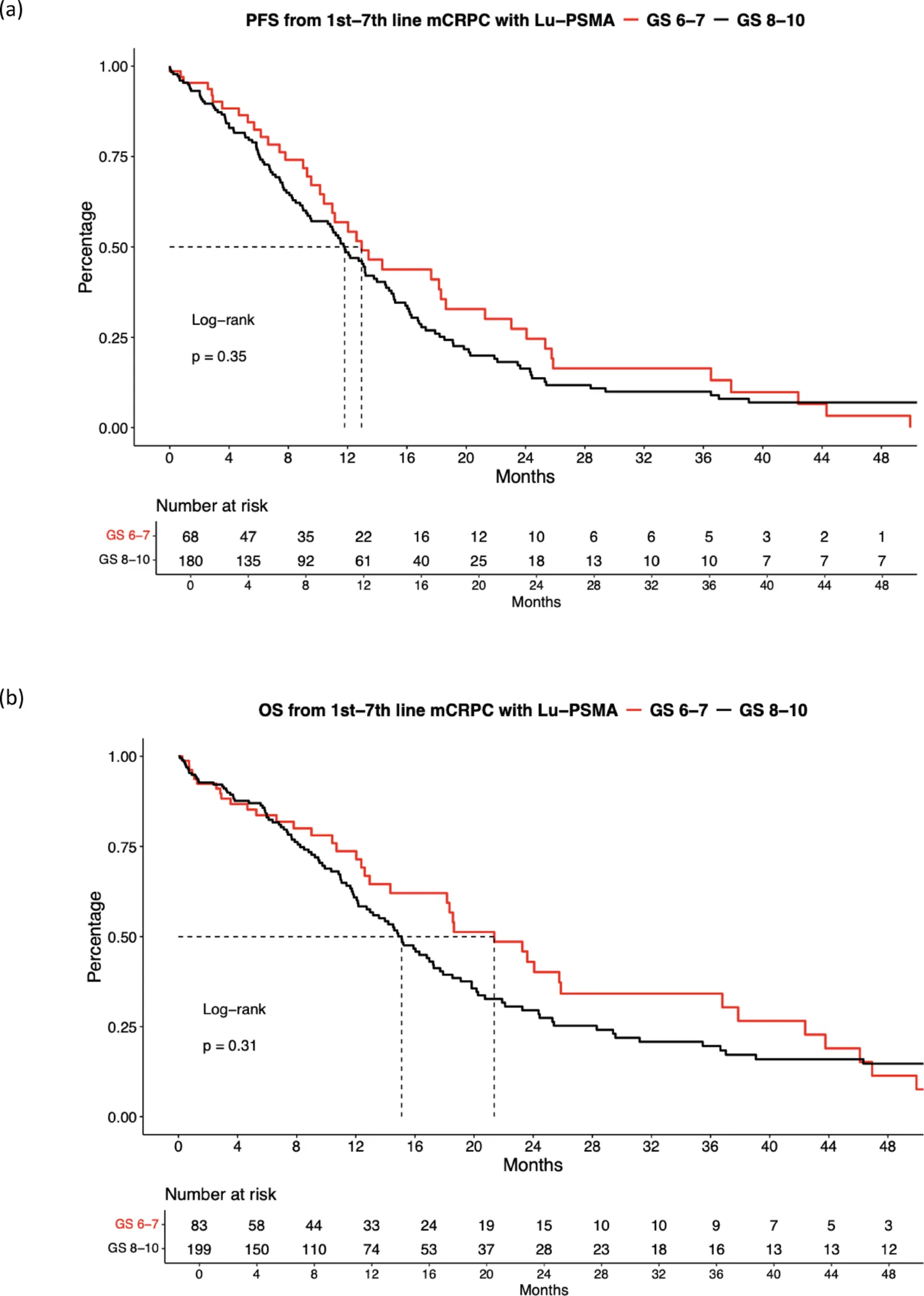
Kaplan Meier curves depicting **a** progression-free survival (PFS) and **b** overall survival (OS) with 177-Lu-PSMA therapy in metastatic castration prostate cancer according to Gleason Score

**Fig. 3:**
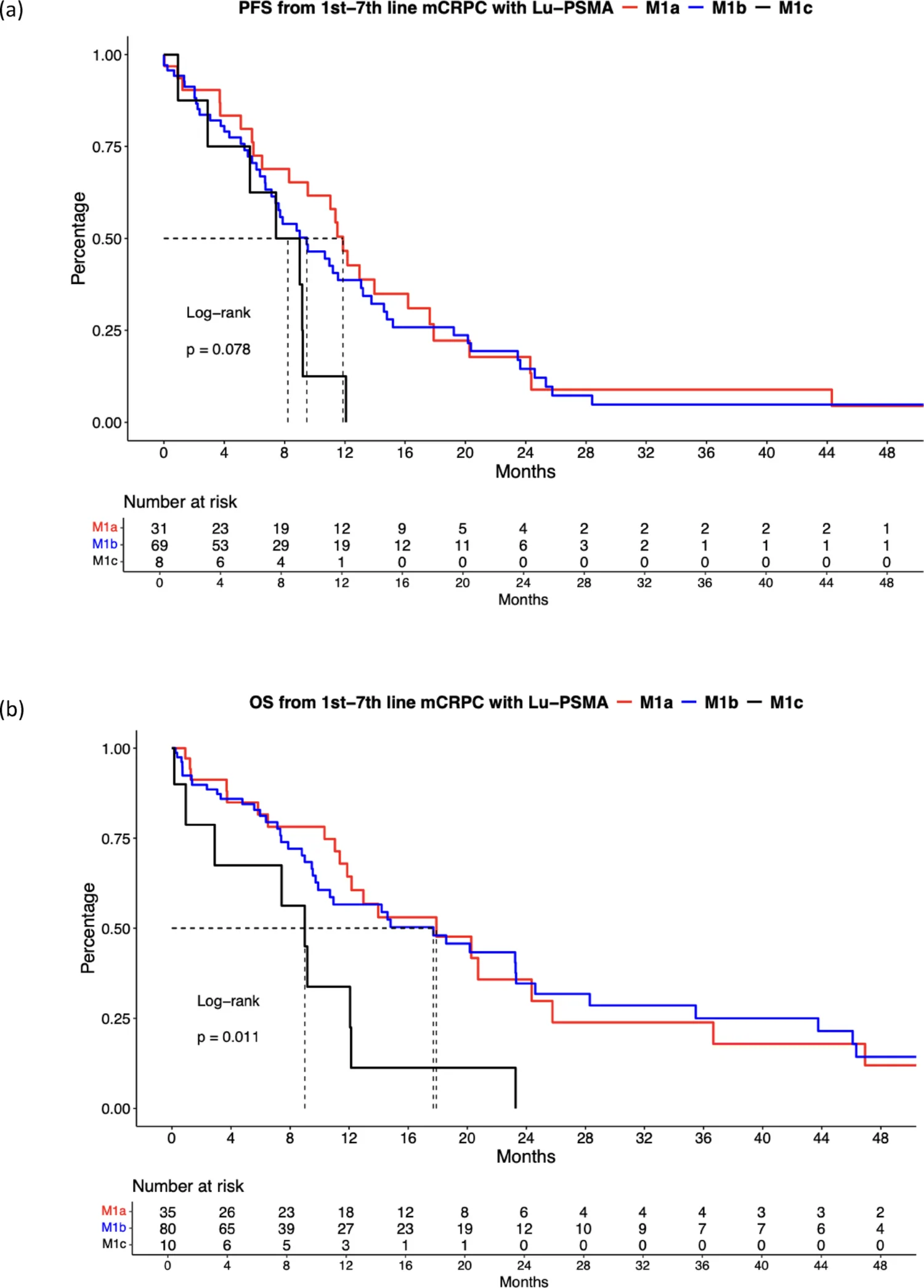
Kaplan Meier curves depicting **a** progression-free survival (PFS) and **b** overall survival (OS) with 177-Lu-PSMA therapy in metastatic castration prostate cancer according to M-stage

Within patients who received Lu-177-PSMA in first to seventh line of therapy for mCRPC (Fig. 1A), no differences were found according to synchronous vs. metachronous patients with median PFS 12.1 vs. 13.0 months (*p* = 0.53). Similarly, no PFS differences were found between GS 6–7 vs. GS8-10 (Fig. 2A) with median PFS 12.9 vs. 11.8 months (*p* = 0.35). According to M-stage, M1a and M1b patients did not harbor significantly more favorable PFS compared to M1c patients (Fig. 3A) with respective median PFS 11.9 vs. 9.5 vs. 8.2 months (*p* = 0.08).

In univariable Cox regression models (Table 4), M1c-stage was significantly associated with worse PFS relative to M1a-stage (HR 2.50, 95% CI 1.10–5.69, *p* = 0.03). After multivariable adjustment including ECOG status and treatment line, synchronous status, GS 8–10, M1b-stage, and M1c-stage were not independently associated with PFS. Compared with Lu-177-PSMA in 1–2 L, later treatment showed numerical associations with shorter PFS that did not reach statistical significance (3 L: HR 1.88, 95% CI 0.97–3.67, *p* = 0.06; 4–7 L: HR 1.97, 95% CI 0.93–4.17, *p* = 0.08).

**Table 4.**
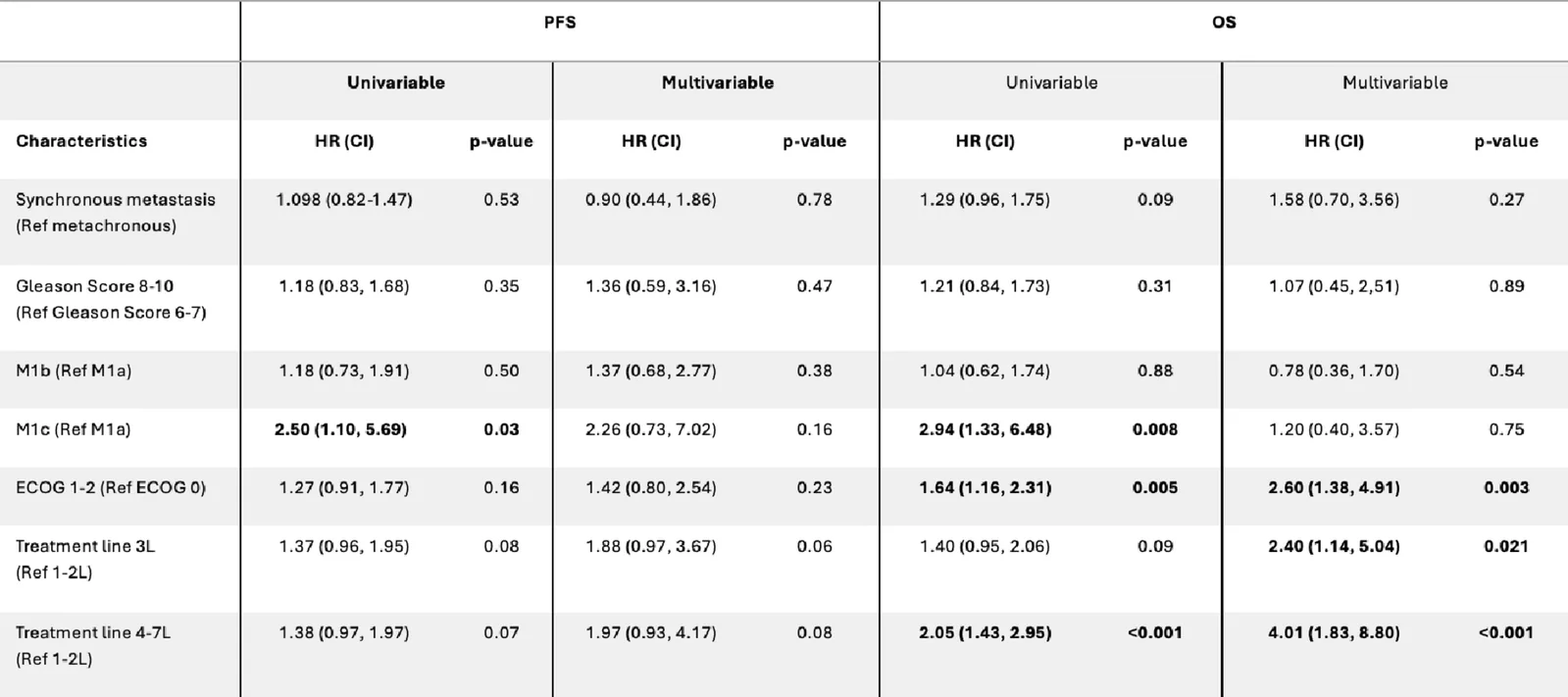
Univariable and multivariable Cox regression models for progression-free survival (PFS) and overall survival (OS) in 344 patients with metastatic castration-resistant prostate cancer patients undergoing 177-Lu-PSMA as first to seventh line after development of castration resistance

### OS according to baseline characteristics

Within patients who received Lu-177-PSMA in first to seventh line of mCRPC (Fig. 1B), no statistically significant difference was observed between synchronous and metachronous patients, with median OS of 14.9 vs. 18.3 months (*p* = 0.09). Similarly, no statistically significant OS difference was observed between GS 6–7 and GS 8–10 (Fig. 2B), with median OS of 21.4 vs. 15.1 months (*p* = 0.31). According to M-stage, median OS was 17.9 vs. 17.7 vs. 9.0 months for M1a, M1b, and M1c disease, respectively (*p* = 0.01; Fig. 3B).

In univariable Cox regression models, M1c-stage was associated with worse OS relative to M1a-stage (HR 2.93, 95% CI 1.33–6.48, *p* = 0.008). After multivariable adjustment including ECOG status and treatment line, synchronous status, GS 8–10, M1b-stage, and M1c-stage were not independently associated with OS. ECOG 1–2 was independently associated with worse OS (HR 2.60, 95% CI 1.38–4.91, *p* = 0.003). Treatment line was also independently associated with OS: compared with 1–2 L, HRs were 2.40 (95% CI 1.14–5.04, *p* = 0.021) for 3 L and 4.01 (95% CI 1.83–8.80, *p* < 0.001) for 4–7 L (Table 4).

## Discussion

We conducted a retrospective study to evaluate survival outcomes of Lu-177-PSMA radioligand therapy in mCRPC patients based on various baseline characteristics at initial mHSPC. We hypothesized that survival outcomes of Lu-177-PSMA may vary according to patients’ baseline characteristics such as initial timing of metastasis in mHSPC (synchronous vs. metachronous), GS (GS 6–7 vs. 8–10) or M-stage (M1a vs. M1b vs. M1c). Using the FRAMCAP database, we tested this hypothesis and made several noteworthy observations.

First, important differences and findings for baseline patient and tumor characteristics of the current real-world cohort were identified and compared to previously reported cohorts of the VISION and TheraP trials [8, 11]. In the current study cohort, median age was 70 years at time of metastasis and 71 years at time of mCRPC, which is comparable to the Lu-177-PSMA cohorts of the VISION and TheraP studies (median age at study enrollment in mCRPC 70 and 72 years, respectively). Median PSA in the current study cohort was 38 ng/ml (IQR 11, 196) at diagnosis and 16 ng/ml (IQR 5, 60) at time of mCRPC, and thus notably lower than in the VISION and TheraP studies (77 and 94ng/ml). Regarding tumor characteristics, the majority of the current study population harbored GS 8–10 (71%), M1b-stage (81%), CHAARTED high-volume disease (64%) and LATITUDE high-risk disease (68%). In contrast, the cohorts of the VISION and TheraP studies harbored lower proportions of GS 8–10 (58% and 54%, respectively) and a higher proportion of patients with bone metastases only (91% in both studies). According to high-volume and high-risk disease, no direct comparison can be made since this information was not provided in neither study. Additionally, in the current real-world study cohort patients more frequently exhibited ECOG 0 (54%) compared to the TheraP-study (41%), while rates of local therapy in the current study cohort (42%) were comparable to that reported in the VISION study (41%). Lastly, in the current study cohort, median number of therapy lines in mCRPC was 3 (IQR 3,5) while Lu-177-PSMA was received mainly within the first five mCRPC lines (92%). In both, VISION and TheraP studies no detailed reports were made according to the number of therapy lines after either ARPI or taxan as the first two therapy lines.

Second, after stratification according to initial timing of metastasis, further important differences exist between patients with initial synchronous vs. metachronous mHSPC undergoing Lu-177-PSMA treatment. Synchronous patients were younger at time of metastasis (68 vs. 72 years) and at time of mCRPC (70 vs. 74 years), had higher PSA-values at diagnosis (100 vs. 13 ng/ml), more frequently exhibited higher ECOG 1–2 status (52 vs. 37%) and GS 8–10 (84 vs. 52%). Furthermore, synchronous patients exhibited higher rates of M1b-stage (89 vs. 70%), while metachronous exhibited higher rates of M1a-stage (19 vs. 4%). Additionally, synchronous patients more frequently harbored CHAARTED [25] -high-volume disease (73 vs. 39%) and LATITUDE [26]-high-risk disease (77 vs. 38%) relative to their metachronous counterparts. These findings largely align with previous reports comparing synchronous and metachronous patients [27–29]. For instance, Frees et al. reported higher rates of GS ≥ 8 and higher PSA-values in synchronous vs. metachronous patients (287 vs. 44 ng/ml), while synchronous patients were less likely to undergo primary local therapy in the same study [30]. Similarly to the current findings of our study, Gebrael et al. observed higher rates of GS ≥ 8 and ECOG > 1 status in synchronous patients compared to metachronous patients. However, unlike both studies, synchronous patients of the current study were younger at time of metastasis and time of mCRPC.

Third, we addressed cancer-control outcome differences in synchronous vs. metachronous patients and found no statistically significant differences in PFS (12.1 vs. 13.0 months, *p* = 0.53) or OS (14.9 vs. 18.3 months, *p* = 0.09). After multivariable adjustment including ECOG status and treatment line, synchronous metastatic presentation was not independently associated with PFS or OS. Thus, in this cohort, initial timing of metastatic disease did not retain independent prognostic significance at the time of later Lu-177-PSMA treatment. In contrast, previous studies examining survival of synchronous vs. metachronous patients treated with standard-of-care regimens such as chemotherapy or ARPI have reported worse survival outcomes in synchronous patients compared with their metachronous counterparts [28, 29, 31].

Fourth, we tested for survival differences according to GS and found no statistically significant differences between GS 6–7 and GS 8–10 for PFS (12.9 vs. 11.8 months, *p* = 0.35) or OS (21.4 vs. 15.1 months, *p* = 0.31). Although median OS differed numerically, this difference was not statistically significant and should be interpreted cautiously. In multivariable Cox regression models additionally adjusted for ECOG status and treatment line, GS 8–10 was not independently associated with PFS or OS. Similarly, Hartrampf et al. found no association between GS and OS (HR 1.09, *p* = 0.5) in patients receiving Lu-177-PSMA [32].

Finally, we tested for cancer-control differences according to M-stage. Median PFS was 11.9 vs. 9.5 vs. 8.2 months for M1a, M1b, and M1c disease, respectively (*p* = 0.08), whereas median OS was 17.9 vs. 17.7 vs. 9.0 months (*p* = 0.01). In univariable Cox regression models, M1c-stage was associated with worse PFS (HR 2.50, *p* = 0.03) and OS (HR 2.93, *p* = 0.008) relative to M1a-stage. However, after multivariable adjustment including ECOG status and treatment line, neither M1b-stage nor M1c-stage was independently associated with PFS or OS. Given the small M1a and M1c subgroups and the resulting wide confidence intervals, these M-stage findings should be regarded as exploratory rather than evidence of a definitive prognostic effect.

While the current study presents novel findings, several limitations should be considered. These include its retrospective, single-center, observational design and potential residual confounding. Some subgroups, particularly M1a and M1c, were small, resulting in limited statistical precision and wide confidence intervals. Comprehensive genomic profiling, quantitative PSMA-PET parameters, and several other potential prognostic variables were not systematically available and therefore could not be reliably incorporated into the models. Treatment line varied substantially across patients; in response to this concern, treatment line was incorporated into the revised multivariable Cox models. Later treatment line was independently associated with worse OS, while the associations of the historical baseline characteristics of primary interest remained non-significant. Finally, PSMA-PET/CT screening before Lu-177-PSMA and multidisciplinary treatment selection may have introduced selection bias.

Taken together, the revised analyses indicate that, among patients treated with Lu-177-PSMA, initial timing of metastatic disease, GS, and M-stage were not independently associated with PFS or OS after adjustment for ECOG status and treatment line. In contrast, later treatment line was independently associated with worse OS, and ECOG 1–2 emerged as an independent adverse prognostic factor for OS. These observational findings describe prognostic associations within a treated cohort and do not establish equivalent efficacy of Lu-177-PSMA across subgroups.

## Conclusion

In this real-world cohort of mCRPC patients treated with Lu-177-PSMA, initial timing of metastatic disease, Gleason score, and M-stage were not independently associated with PFS or OS after multivariable adjustment including ECOG status and treatment line. These findings are exploratory and should not be interpreted as evidence of equivalent treatment efficacy across subgroups.

## Data Availability

The datasets generated during and/or analyzed during the current study are available from the corresponding author on reasonable request.
